# Trends and inequity in improved sanitation facility utilisation in Bangladesh: Evidence from Bangladesh Demographic and Health Surveys

**DOI:** 10.1186/s13104-023-06555-0

**Published:** 2023-10-31

**Authors:** Anisuddin Ahmed, Abu Sayeed, Tania Sultana Tanwi, Nondo Saha, Molly Hanson, Dipanjan Adhikary Protyai, Aniqa Tasnim Hossain, Ali Ahmed, Fariya Rahman, Ema Akter, Nowrin Nusrat, Md Shawon Badsha, Afruna Rahman, Md Khairul Islam, Md. Shah Alam, Quamrun Nahar, Shams El Arifeen, Ahmed Ehsanur Rahman, Tazeen Tahsina

**Affiliations:** 1 Department of Women’s and Children’s Health, Uppsala University, 75205 Uppsala, Sweden; 2 Maternal and Child Health Division (MCHD), International Centre for Diarrhoeal Disease Research (icddr,b), Dhaka, Bangladesh; 3 Institute of Statistical Research and Training, University of Dhaka, Dhaka, Bangladesh; 4 Western Sydney University, Penrith Campus, Sydney, Australia; 5 Infectious Disease Division (IDD), International Centre for Diarrhoeal Disease Research (icddr,b), Dhaka, Bangladesh; 6 WaterAid, Dhaka, Bangladesh

**Keywords:** Bangladesh, BDHS, Improved sanitation, Trends, Equity

## Abstract

Improved sanitation is indispensable to human health. However, lack of access to improved sanitation remains one of the most daunting public health challenges of the twenty-first century in Bangladesh. The aim of the study was to describe the trends in access to improved sanitation facilities following the inequity gap among households in different socioeconomic groups in Bangladesh. Data from the Bangladesh Demographic and Health Survey (BDHS) 2007, 2011, 2014, and 2017-18 were extracted for this study. Inequity in access to improved sanitation was calculated using rich-poor ratio and concentration index to determine the changes in inequity across the time period. In Bangladesh, the proportion of households with access to improved sanitation increased steadily from 25.4% to 45.4% between 2007 and 2014, but slightly decreased to 44.0% in 2017-18. Age, educational status, marital status of household head, household wealth index, household size, place of residence, division, and survey year were significantly associated with the utilisation of improved sanitation. There is a pro-rich situation, which means that utilisation of improved sanitation was more concentrated among the rich across all survey years (Concentration Index ranges: 0.40 to 0.27). The government and other relevant stakeholders should take initiatives considering inequity among different socioeconomic groups to ensure the use of improved sanitation facilities for all, hence achieving universal health coverage.

**Supplementary Information:**

The online version contains supplementary material available at 10.1186/s13104-023-06555-0.

## Introduction

Access to improved sanitation is a basic human right for every person [1, 2]. As defined by the WHO/UNICEF Joint Monitoring Programme  (JMP) on Water Supply, Sanitation, and Hygiene (WASH), ‘improved’ sanitation facilities are not shared with other households and are designed to hygienically separate excreta from human contact [3]. However, nearly one-third of the global population, over 3.6 billion people, still do not have access to this fundamental need [3, 4].

Unimproved sanitation perpetuates a high risk of disease transmission, including cholera, typhoid, schistosomiasis, respiratory infections, skin infections, eye infections, and even certain cancers due to exposure to carcinogens [5]. Moreover, it also increases the burden of malnutrition [6]. Therefore, poor sanitation significantly contributes to a high transmission of neglected tropical diseases in low-and middle-income countries (LMICs) and around 432,000 deaths annually [7].

Improved sanitation is considered one of the most significant public health needs which requires much attention in LMICs. Currently, around 27.0% of the population in LMICs has access to improved sanitation [8]. Rural communities within LMICs make up the majority of the population who do not have access to basic sanitation and approximately 90.0% practice open defecation [8]. In Bangladesh, less than half of the population (47.0%) has access to basic sanitation in 2015 [3]. Between 2006 and 2009, there was an almost two-fold increase in the availability of improved sanitation facilities, with both rural and urban regions exhibiting remarkable growth [9]. Despite a variety of updated sanitation interventions, the coverage of improved sanitation could not reach the optimal level across all socioeconomic groups in Bangladesh [10].

Studying the determinants associated with the utilisation of improved sanitation is one of the ways of understanding these inequities among people with different socioeconomic status. Evidence suggests that wealth index, gender, age and education of household head, and household size are associated with the utilisation of improved sanitation in LMICs [11, 12]. A limited number of studies have been conducted on improved sanitation facilities in Bangladesh [9, 10, 13, 14]. Moreover, a few studies have covered the comprehensive picture of improved sanitation utilisation after the country-wide interventions [15, 16]. Therefore, the objective of this study is to investigate the trend of utilisation of improved sanitation, its associated factors and persisting inequities in service utilisation in Bangladesh.

### Methods

#### Data source

Data were extracted from four rounds of Bangladesh Demographic and Health Surveys (BDHS) during 2007, 2011, 2014, and 2017-18. These nationally representative surveys covered information on socio-demographic and -economic characteristics, family planning, utilisation of maternal and child health services, and access to water and sanitation [17–20].

#### Study population

These cross-sectional surveys followed a two-stage stratified random sampling of households [17–20]. The BDHSs of 2007, 2011, 2014, and 2017-18 collected information from 10,400, 17,141, 17,300, and 19,457 households, respectively (**Supplementary Fig. 1**). Rangpur division, formed in 2010 as Bangladesh’s 7th division, was basically a part of Rajshahi division and Mymensingh, the 8th administrative division of Bangladesh, was composting the northern part of Dhaka division before 2015 [21]. Therefore, data from Rangpur was included in Rajshahi division in 2007 and similarly, Mymensingh data was included in Dhaka division in 2007, 2011, and 2014. To keep homogeneity, we combined Rangpur division and Rajshahi division as ‘Rajshahi division’ for BDHS 2011, 2014, 2017-18 and Mymensingh division and Dhaka division as ‘Dhaka division’ for BDHS 2017-18.

#### Variable description

In this study, we have defined improved sanitation (basic) according to JMP by WHO and UNICEF as follows: an improved sanitation facility is “one that hygienically separates human excreta from human contact” and that is not shared with other households [3]. Improved sanitation facilities include: flush or pour-flush to piped sewer system, septic tank or pit latrine; ventilated improved pit latrine; pit latrine with slab and composting toilet. However, sanitation facilities are not considered improved when shared with other households, or open to public use. While, unimproved sanitation facilities include: flush or pour-flush to elsewhere; pit latrine without slab or open pit; bucket, hanging toilet or hanging latrine, bush or field (open defecation) [3]. In our data, we had two variables which represents toilet facility and shared status of toilet. Following the definition, we recoded these two variables as “1” for improved toilet facility and “0” for not improved toilet facility and shared status as “1” for not shared and “0” for shared toilet. Finally, we created a new variable with two categories, “1” indicating improved sanitation (both improved toilet facility and not shared toilet) and “0” indicating not improved sanitation (either not improved toilet facility or shared toilet). We have used this variable as our dependent variable.

### Statistical analysis

#### Descriptive analysis and logistic regression

The statistical analysis was conducted using STATA 16.0 (Stata, College Station, TX, USA). Sampling weight was adjusted while performing the analysis. Initially, descriptive analyses were performed to describe the trends in access to improved sanitation facilities over time. Proportion and chi-square tests were done according to socio-demographic and -economic characteristics of the households. We performed binary logistic regression modelling with 95% confidence intervals and tests of statistical significance using pooled data of the four consecutive surveys to determine the factors associated with improved sanitation access. The covariates controlled in the adjusted model are the division, place of residence, wealth index, age, marital status, education level of the household head, and survey years. Adjusted odds ratio (AOR) with 95% confidence intervals (CIs) was presented and p-value < 0.05 was considered statistically significant during the regression analysis. The concentration indices for inequity measurement was calculated for utilisation of improved sanitation and the household’s wealth score. These two estimates were then plotted to generate concentration curves and observe any changes in inequity over the time period.

### The operational definition of concentration curves and index

The concentration index is a useful tool proposed by the World Health Organization for assessing the degree of equity of health-related indicators in different economic and social contexts [22]. The concentration curve delineates inequity by plotting the cumulative percentage of improved sanitation utilisation with respect to the cumulative percentage of the household’s wealth score. When the concentration curve conforms to the line of equity at 45°, it indicates perfect equity. A curve that lies above the perfect equity line means the improved sanitation utilisation is concentrated among the poor, and vice versa. The concentration index gives the magnitude of inequity, ranging from − 1 to + 1, and is also defined as twice the area between the concentration curve and the line of equity. Perfect equity is achieved when the index value is zero; the index value closer to -1 means disproportionate improved sanitation utilisation among poor households, while the concentration of improved sanitation utilisation increases among the rich if the index value is closer to +1 [23]. Wagstaff developed a modified concentration index by re-scaling the standard index to keep unscathed the relative inequity variance property of the concentration index [22]. For the corrected concentration index for this study, “*conindex”* command of STATA has been used [24].

## Results

Table 1 shows that improved sanitation utilisation increased from 25.4 to 44.0% from 2007 to 2017-18. Table 1 also demonstrates the distribution of using improved sanitation facilities among households by socio-demographic and -economic backgrounds across the time periods.

**Table 1:** Percentage distribution of household sanitation facility by background characteristics of households

Background characteristics	2007 (n = 10,400)			2011 (n = 17,141)			2014 (n = 17,300)			2017-18 (n = 19,457)		
	Improved	Not improved	*p*-value	Improved	Not improved	*p*-value	Improved	Not improved	*p*-value	Improved	Not improved	*p*-value
Division												
Barisal	33.51	66.49	0.009	38.40	61.60	< 0.001	52.01	47.99	< 0.001	47.83	52.17	< 0.001
Chittagong	29.77	70.23		38.50	61.50		53.06	46.94		54.09	45.91	
Dhaka	22.95	77.05		29.03	70.97		41.55	58.45		39.65	60.35	
Khulna	27.35	72.78		35.51	64.49		47.53	52.47		49.82	50.18	
Rajshahi	22.22	77.78		33.74	66.26		43.26	56.74		39.44	60.56	
Sylhet	26.68	73.32		36.54	63.46		42.52	57.48		44.78	55.22	
Place of residence												
Urban	37.83	62.17	< 0.001	39.57	60.43	< 0.001	49.76	50.24	0.007	45.26	54.74	0.354
Rural	21.99	78.01		31.67	68.33		43.65	56.35		43.47	56.53	
Wealth Index												
Poorest	6.80	93.20	< 0.001	8.80	91.20	< 0.001	18.38	81.62	< 0.001	17.02	82.98	< 0.001
Poorer	12.72	87.28		22.76	77.24		35.48	64.52		30.97	69.03	
Middle	20.40	79.60		33.46	66.54		50.70	49.30		50.25	49.75	
Richer	34.06	65.94		46.55	53.45		53.84	46.16		51.07	48.93	
Richest	57.22	42.78		60.83	39.17		69.28	30.72		73.00	27.00	
Household size (Members)												
1–3	16.67	83.33	< 0.001	23.46	76.54	< 0.001	35.81	64.19	< 0.001	34.84	65.16	< 0.001
4–6	25.07	74.93		35.07	64.93		46.91	53.09		46.00	54.00	
7 or more	37.46	62.54		44.48	55.52		57.43	42.47		56.22	43.78	
Sex of the household head												
Male	25.48	74.52	0.821	33.77	66.23	0.413	45.08	54.92	0.206	44.17	55.83	0.327
Female	25.14	74.86		32.68	67.32		47.33	52.67		42.95	57.05	
Age of household head (Years)												
Less than 30	14.30	85.70	< 0.001	19.63	80.37	< 0.001	26.07	73.93	< 0.001	29.09	70.91	< 0.001
30–39	20.19	79.81		27.95	72.05		39.50	60.50		38.64	61.36	
40–49	29.89	70.11		37.53	62.48		49.83	50.17		48.00	52.00	
50 or more	30.92	69.08		39.99	60.01		53.47	46.53		49.57	50.43	
Educational status of household head												
No education	14.28	85.72	< 0.001	22.08	77.92	< 0.001	33.54	66.46	< 0.001	31.86	68.14	< 0.001
Primary	21.79	78.21		29.47	70.53		40.73	59.27		38.75	61.25	
Secondary	34.51	65.49		42.66	57.34		53.63	46.37		50.43	49.57	
Higher	62.72	37.28		63.69	36.31		74.32	25.68		73.16	26.84	
Ever married status of household head												
No	28.14	71.86	0.448	29.55	70.45	0.113	45.98	54.02	0.857	42.81	57.19	0.735
Yes	25.37	74.63		33.75	66.25		45.36	54.64		43.99	56.01	
Overall	25.44	74.56		33.65	66.35		45.36	54.64		43.97	56.03	

The covariates, including age, educational status, and marital status of household head, wealth index, household size, division, place of residence, and survey years were significantly associated with utilisation of improved sanitation (Table 2). Households with a head aged ≥ 50 years were 2.7 times more likely to practice improved sanitation than those < 30 years (AOR = 2.73, 95% CI = 2.56–2.93). The household heads with higher education had 3.0 (AOR = 2.96, 95% CI = 2.76–3.17) times higher likelihood of using improved sanitation compared to non-educated household heads. Ever married household heads were 49% more likely to have access of improved sanitation than individuals who were not married. The richest households were 5.0 times (AOR = 5.04, 95% CI = 4.30–5.91) more likely to have access of improved sanitation as compared to lower socioeconomic status households. Compared to the capital Dhaka division, households situated in the Barishal division were 2.2 times (AOR = 2.20, 95% CI = 2.03–2.38) more likely to have access to improved sanitation. Rural households were 37% less likely (AOR = 0.63, 95% CI = 0.53–0.73) to use improved sanitation compared to urban households.

**Table 2:** Crude odds ratios (COR) and adjusted odds ratios (AOR) of households using improved sanitation and their 95% confidence interval, Bangladesh (N = 64,298)

Background characteristics	Crude Odds Ratio (95% CI)	*p*-value	Adjusted Odds Ratio (95% CI)	*p*-value
Division (Ref: Dhaka)				
Chittagong	1.47 (1.37–1.57)*	< 0.001	1.61 (1.53–1.70)*	< 0.001
Barishal	1.46 (1.37–1.57)*	< 0.001	2.20 (2.03–2.38)*	< 0.001
Khulna	1.31 (1.24–1.39)*	< 0.001	1.50 (1.41–1.59)*	< 0.001
Rajshahi	1.05 (1.01–1.10)*	0.024	1.58 (1.50–1.66)*	< 0.001
Sylhet	1.20 (1.12–1.29)*	< 0.001	1.37 (1.26–1.49)*	< 0.001
Place of residence (Ref: Urban)				
Rural	0.73 (0.71–0.76)*	< 0.001	0.63 (0.53–0.73)*	< 0.001
Wealth Index (Ref: Poorest)				
Poorer	2.38 (2.24–2.54)*	< 0.001	1.53 (1.25–1.88)*	< 0.001
Middle	4.48 (4.22–4.76)*	< 0.001	1.72 (1.43–2.05)*	< 0.001
Richer	5.95 (5.60–6.32)*	< 0.001	1.77 (1.50–2.08)*	< 0.001
Richest	12.67 (11.91–13.48)*	< 0.001	5.04 (4.30–5.91)*	< 0.001
Household size (Ref: 1–3)				
4–6	1.58 (1.52–1.64)*	< 0.001	1.58 (1.51–1.65)*	< 0.001
7 or more	2.32 (2.20–2.44)*	< 0.001	2.03 (1.91–2.15)*	< 0.001
Age of household head (Ref: Less than 30)				
30–39 Years	1.64 (1.55–1.75)*	< 0.001	1.48 (1.38–1.58)*	< 0.001
40–49 Years	2.47 (2.33–2.62)*	< 0.001	2.31 (2.15–2.48)*	< 0.001
50 or more years	2.77 (2.62–2.93)*	< 0.001	2.73 (2.56–2.93)*	< 0.001
Educational status of household head (Ref: No education)				
Primary	1.48 (1.42–1.54)*	< 0.001	1.22 (1.17–1.28)*	< 0.001
Secondary	2.51 (2.40–2.62)*	< 0.001	1.57 (1.50–1.66)*	< 0.001
Higher	6.45 (6.00–6.83)*	< 0.001	2.96 (2.76–3.17)*	< 0.001
Marital status of household head/Ever married (Ref: Yes)				
No	0.92 (0.82–1.04)	0.181	1.49 (1.29–1.72)*	< 0.001
Study point/Period (Ref: year 2007)				
2011	1.49 (1.41–1.57)*	< 0.001	1.67 (1.57–1.78)*	< 0.001
2014	2.43 (2.31–2.57)*	< 0.001	3.17 (2.98–3.37)*	< 0.001
2017-18	2.30 (2.18–2.42)*	< 0.001	2.95 (2.78–3.13)*	< 0.001

*p < 0.05

**Fig. 1:**
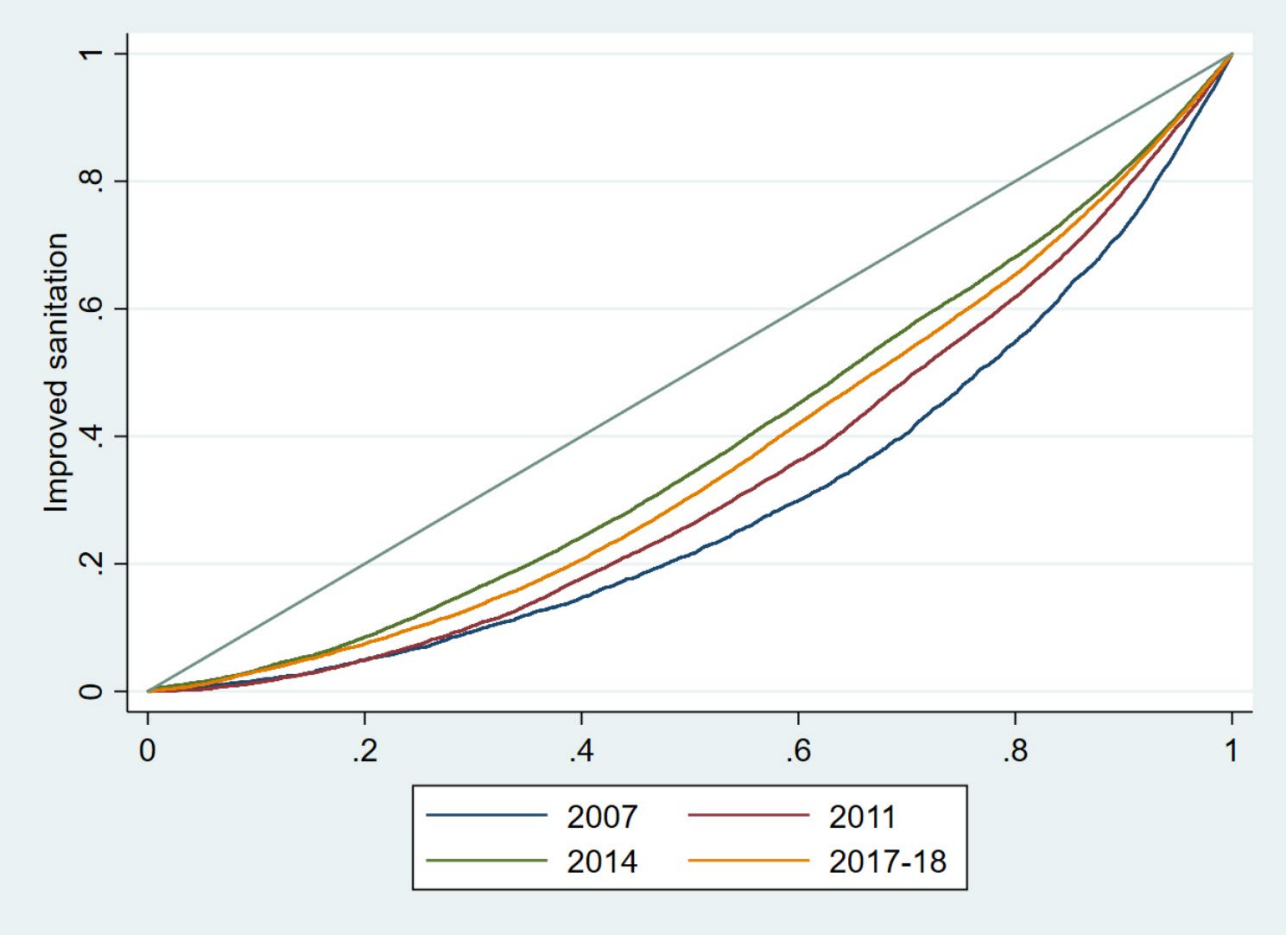
Equity distribution of improved sanitation utilisation over the time from 2007 to 2017-18

The rich-poor ratio for utilisation of improved sanitation reduced from 8.4:1 to 4.3:1 between 2007 and 2017-18 (Table 1) and the concentration index also declined from 0.40 to 0.27 (p < 0.001) (**Supplementary Table 1)**. Figure 1 shows the concentration curves of utilisation of improved sanitation from 2007 to 2017-18. The situation was pro-rich, indicating the use of improved sanitation was more concentrated among the rich in the years 2007, 2011, 2014, and 2017-18.

## Discussion

This study has revealed that the utilisation of improved sanitation is considerably increasing. Though the rich-poor gap reduced over the time, the inequity is still prevalent between poor and rich communities in accessing improved sanitation. Improved sanitation was better utilised by the households, emphasised by several socio-demographic and -economic factors such as age, educational status, and marital status of household head, wealth index, household size, division, place of residence, and over the time periods.

This study shows that Bangladesh has made consistent positive progress in improving sanitation facilities from 2007 (25.4%) to 2014 (45.4%) [17–20, 25], similar to studies conducted in other developing countries [11, 12, 25–27]. However, this study also found that improved sanitation facility utilisation decreased in 2017-18 (44.0%) compared to 2014 (45.4%). In this unexpected downturn of improved sanitation utilisation, the risk of waterborne diseases such as cholera, diarrhoea, and dysentery, as well as sexual and reproductive health conditions could be increased [28–31].

Our findings show that wealthy households were more likely to use improved sanitation facilities compared to poorer households, and these results are similar to the previous studies [11, 28, 32]. The plausible explanation is that wealthier people have more ability to pay for improved sanitation [33–35].

The Government of Bangladesh has undertaken multiple programmes to promote improved sanitation at all socioeconomic levels of households since 2006. This study found inequity is still existing in 2017-18. Although the rich-poor gap declined consistently from 2007 to 2014 (8.3:1 to 3.8:1), it started to increase (4.3:1) again by 2017-18. Therefore, to achieve universal health coverage by 2030, it is imperative to ensure equitable sanitation facilities for all. A range of studies highlighted that unless governments and relevant stakeholders adopt strategies deliberately targeting all socioeconomic population groups, it would be challenging to achieve universal coverage [36–38].

Households with household heads who had achieved higher education were more likely to have access to improved sanitation than their counterparts. This finding is consistent with other similar studies [39–42]. The finding can be attributed to educated household heads having more knowledge of the health risks associated with poor sanitation systems [43]. Residential differences (living in rural or urban areas) in access to improved sanitation have been observed in this study. Result shows that people from rural areas had lower odds (AOR = 0.63, 95% CI: 0.53–0.73) of using improved sanitation. This was expected, as prior studies found a similar result [5, 28, 32]. Flooding occurs on an annual basis in approximately one-third of Bangladesh, while other areas of the country experience seasonal water shortages [14]. The capacity of rural Bangladeshis to construct and maintain latrines are impacted by each of these factors to varying degrees [14]. To improve this scenario, improved sanitation programmes were likely focused on rural areas [14], reflected in the proportion of utilisation among the rural residents considerably increasing by 2017-18 as compared to urban residents.

The age of the household head was positively correlated with improved sanitation utilisation. The possible explanation could be that older household heads have more knowledge regarding the importance of improved sanitation [12]. Our findings also show significant divisional variation in this study. Compared to the Dhaka division, all other divisions have a higher probability of using improved sanitation. This can be attributed to the high number of people living in slum areas of Dhaka division with inadequate sanitation [44–46].

### Strengths and limitations

This study has several strengths. Firstly, using large nationally representative surveys’ data that were conducted at different periods. Secondly, the response rate of the participants was excellent. The limitation of this study was that the data in the BDHS was acquired using cross-sectional methods, which restricted the potential for drawing causal inferences.

### Conclusion and recommendation

Bangladesh has made significant progress in accessing improved sanitation facilities over the years, but the disparity the between rich and poor remains a matter of concern. Since the proportion of the households using improved sanitation facilities remained low in this study, greater progress is needed for the poorest households. Further research should focus on the community demands to improve sanitation facilities and overcome barriers to achieve progress. Despite this, the existing wealth inequity in accessing the improved sanitation could be reduced by adopting integrated intervention approaches involving both the community and the local government authorities. These findings also suggest that governmental and non-governmental organisations should take initiatives on WASH, considering multifaceted policy strategies that account for regional and residence differences, as well as other defined factors, to achieve universal health coverage in Bangladesh. Moreover, in-depth qualitative research is required to better articulate the recent decline in utilisation of the improved sanitation services in Bangladesh.

### Electronic supplementary material

Below is the link to the electronic supplementary material.

Supplementary Material 1

## Data Availability

All data are publicly available upon registration in DHS program supported by USAID at https://dhsprogram.com/data/available-datasets.cfm?ctryid=1. Specifically, the minimal data used for this study are available from the corresponding author on reasonable request.

## List of abbreviations

AOR: Adjusted odds ratio
BDHS: Bangladesh Demographic and Health Survey
Cis: Confidence intervals
icddr,b: International Centre for Diarrhoeal Disease Research, Bangladesh
JMP: Joint Monitoring Programme
LMICs: Low-and middle-income countries
OR: Odds ratio
UNICEF: United Nations Children’s Fund
WHO: World Health Organization
