# Supplementary material for: Trends and inequity in improved sanitation facility utilisation in Bangladesh: Evidence from Bangladesh Demographic and Health Surveys
Source: BMC Res Notes. 2023 Oct 31;16:303. doi: 10.1186/s13104-023-06555-0 (PMC10619219; doi:10.1186/s13104-023-06555-0)
Supplement: Supplementary file 1 — Supplementary Material 1 [file 13104_2023_6555_MOESM1_ESM.docx]

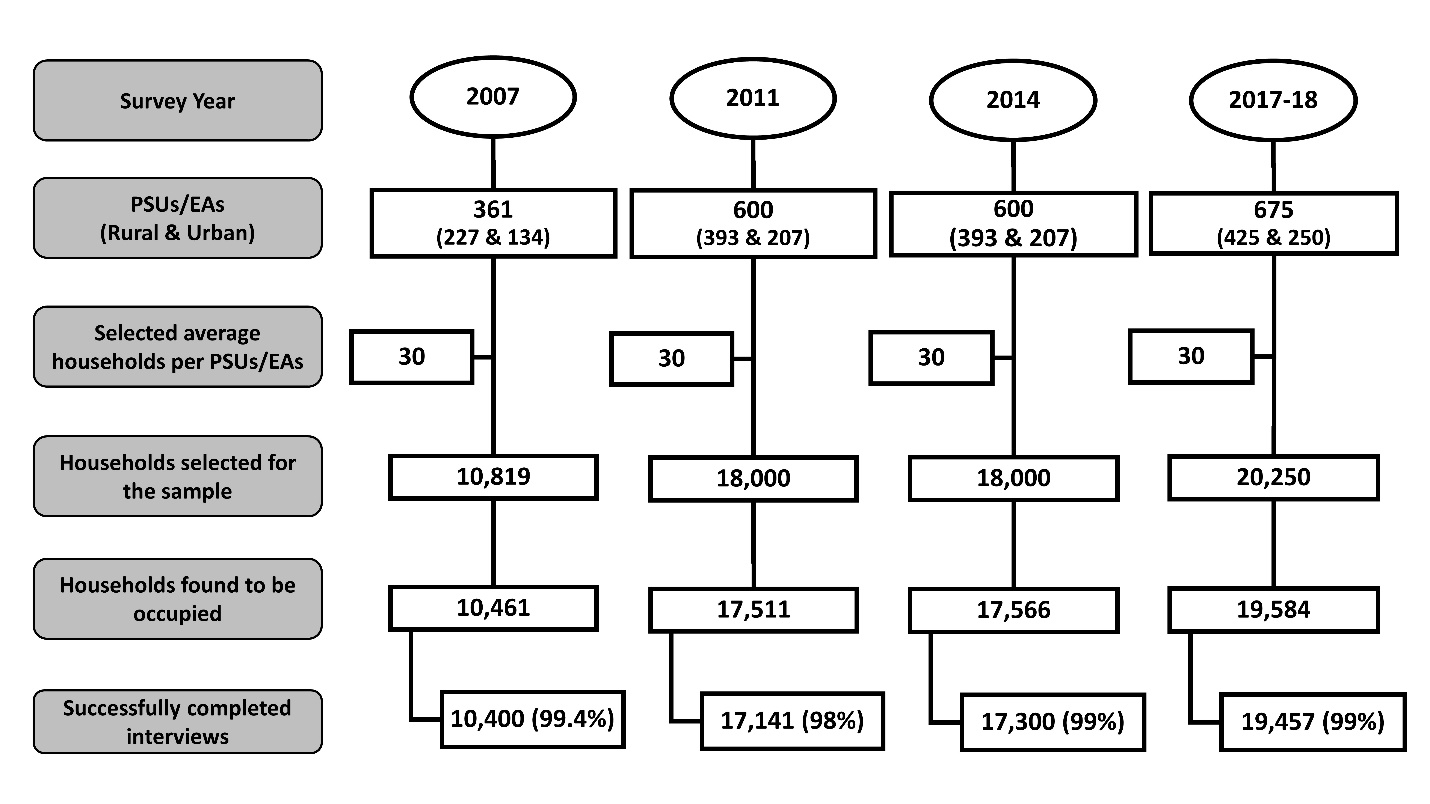


**Supplementary Figure 1: Sampling flowchart for the selection of households for BDHS 2007, 2011, 2014 and 2017-18**

**Supplementary Table 1: Measure of inequality (Concentration Index and 95% Confidence Interval) by survey year**

| Year | Concentration Index (CI) | Standard Error | P value |
| --- | --- | --- | --- |
| 2007 | 0.404 | 0.007 | <0.001 |
| 2011 | 0.333 | 0.005 | <0.001 |
| 2014 | 0.227 | 0 .004 | <0.001 |
| 2017-18 | 0 .271 | 0.004 | <0.001 |
